# Supplementary material for: Ultrasensitive detection of nucleic acids using deformed graphene channel field effect biosensors
Source: Nat Commun. 2020 Mar 24;11:1543. doi: 10.1038/s41467-020-15330-9 (PMC7093535; doi:10.1038/s41467-020-15330-9)
Supplement: Supplementary file 1 — Supplementary Information [file 41467_2020_15330_MOESM1_ESM.pdf]

SUPPLEMENTARY INFORMATION

Ultrasensitive detection of nucleic acids using deformed graphene channel field effect biosensors

Michael Taeyoung Hwang[1,&], Mohammad Heiranian[2,&], Yerim Kim[2,&], Seungyong You[1], Juyoung Leem[2], Amir Taqieddin[2], Vahid Faramarzi[3], Yuhang Jing[4,5], Insu Park[1], Arend M. van der Zande[1,2,7], Sungwoo Nam[2,6,7], Narayana Aluru[2,7*], Rashid Bashir[1,2,3,6,7*]

[1]Holonyak Micro and Nanotechnology Laboratory, University of Illinois at Urbana-Champaign, Urbana, IL, USA

[2]Department of Mechanical Science and Engineering, University of Illinois, Urbana-Champaign, IL, USA

[3]Department of Bioengineering, University of Illinois, Urbana-Champaign, IL, USA

[4]Beckman Institute for Advanced Science and Technology, University of Illinois at Urbana-Champaign, Urbana, Illinois 61801, USA.

[5]Department of Astronautical Science and Mechanics, Harbin Institute of Technology, Harbin, Heilongjiang 150001, P. R. China.

[6]Department of Material Science and Engineering, University of Illinois, Urbana-Champaign, IL, USA

[7]Materials Research Laboratory, University of Illinois, Urbana-Champaign, IL, USA

[&]These authors contributed equally

*e-mail: rbashir@illinois.edu, aluru@illinois.edu

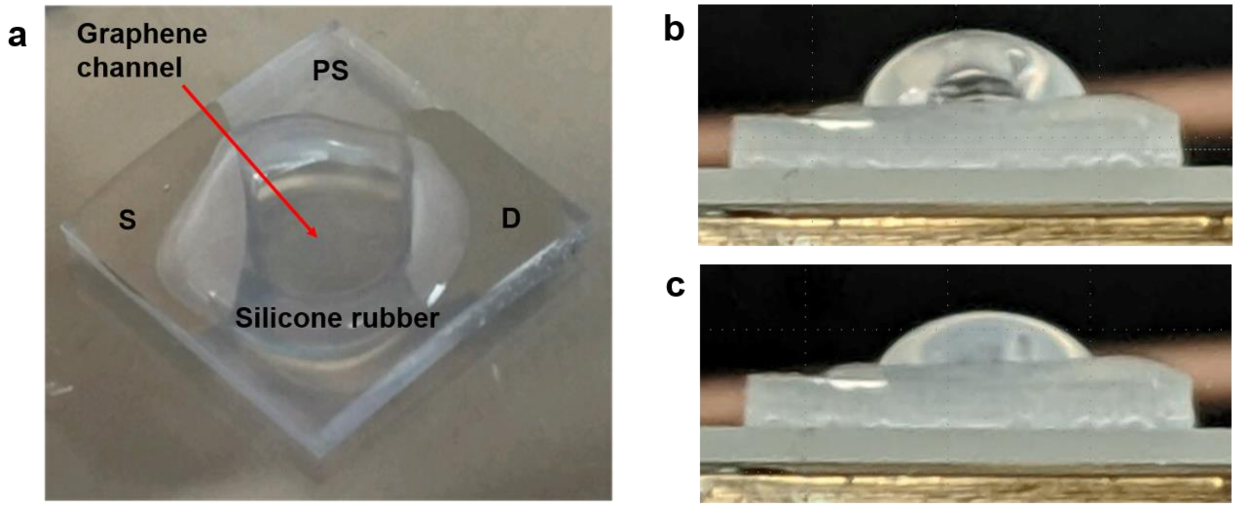

Supplementary Figure 1. Optical image of the crumpled graphene FET. a, Top view of the FET. S is source electrode and D is drain electrode made by silver paste. PS is polystyrene substrate. The liquid chamber was created using a silicone rubber. Graphene channel is indicated with red arrow. b, Lateral view of the device with 50 µl of PBS solution droplet. c, Lateral view of the device with 50 µl of PBS solution droplet after 1 hour in the probe station. ~18 µl of water was dried thus the size of the droplet became smaller.

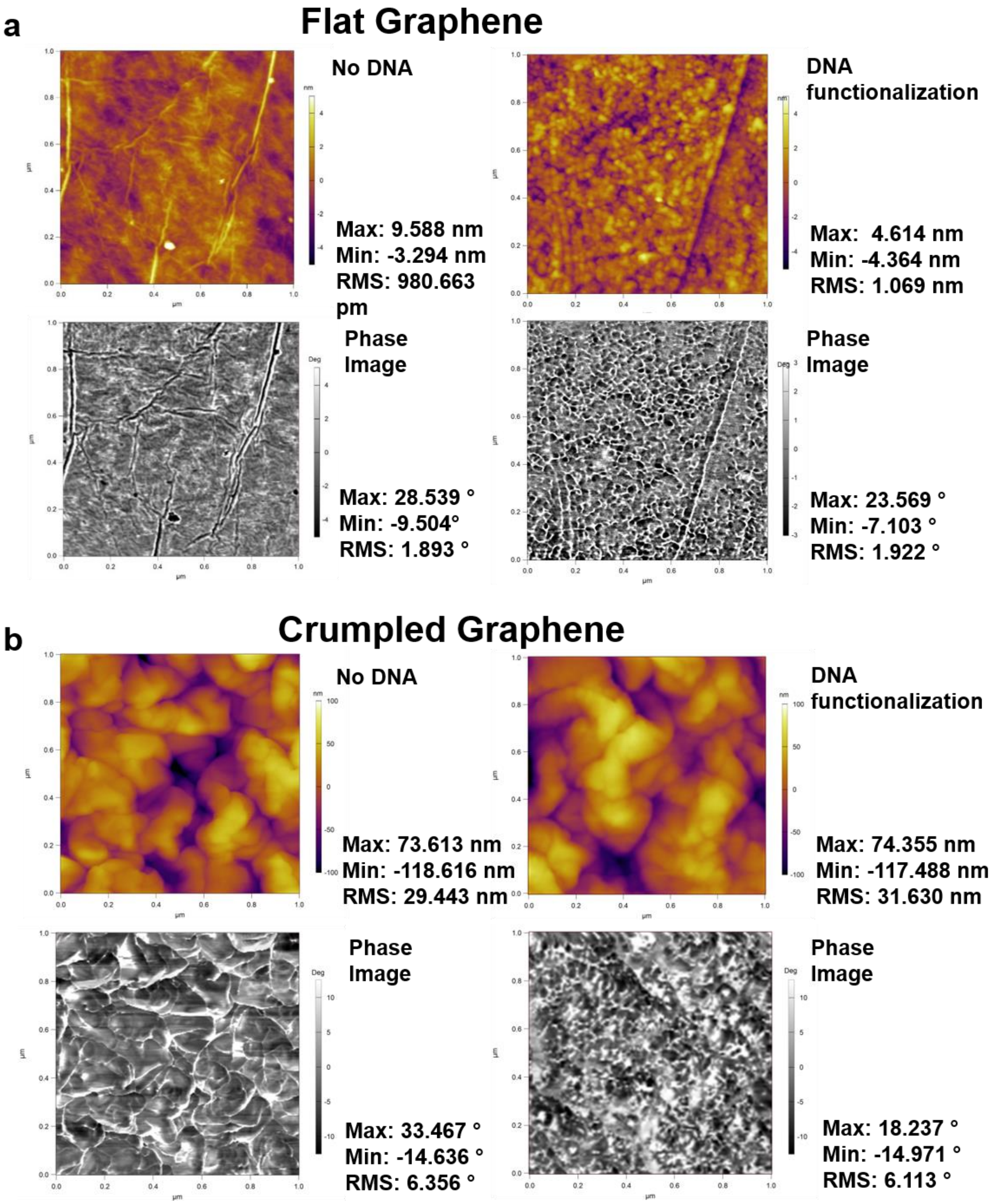

Supplementary Figure 2. AFM images of flat and crumpled graphene transistor surface with and without the DNA immobilization. **a left**, AFM imaging and phase image of flat graphene surface in air showed mostly flat surface with some wrinkles. **a right**, flat graphene surface covered with DNA in air. The strands produce features polygonal structure. **b left**, AFM imaging and phase image of crumpled graphene surface in air. Increased crumple height/roughness is observed. Hierarchical wrinkling is clearly evident. **b right**, in AFM image, significant difference was not observed before and after DNA

functionalization. However, phase imaging showed polygonal structure which was similar to the DNA features on flat graphene. For AFM imaging, 47 nt of partially double stranded DNA was used (Supplementary Table 1). All images have a scan area of $1 \times 1\ \mu m^2$.

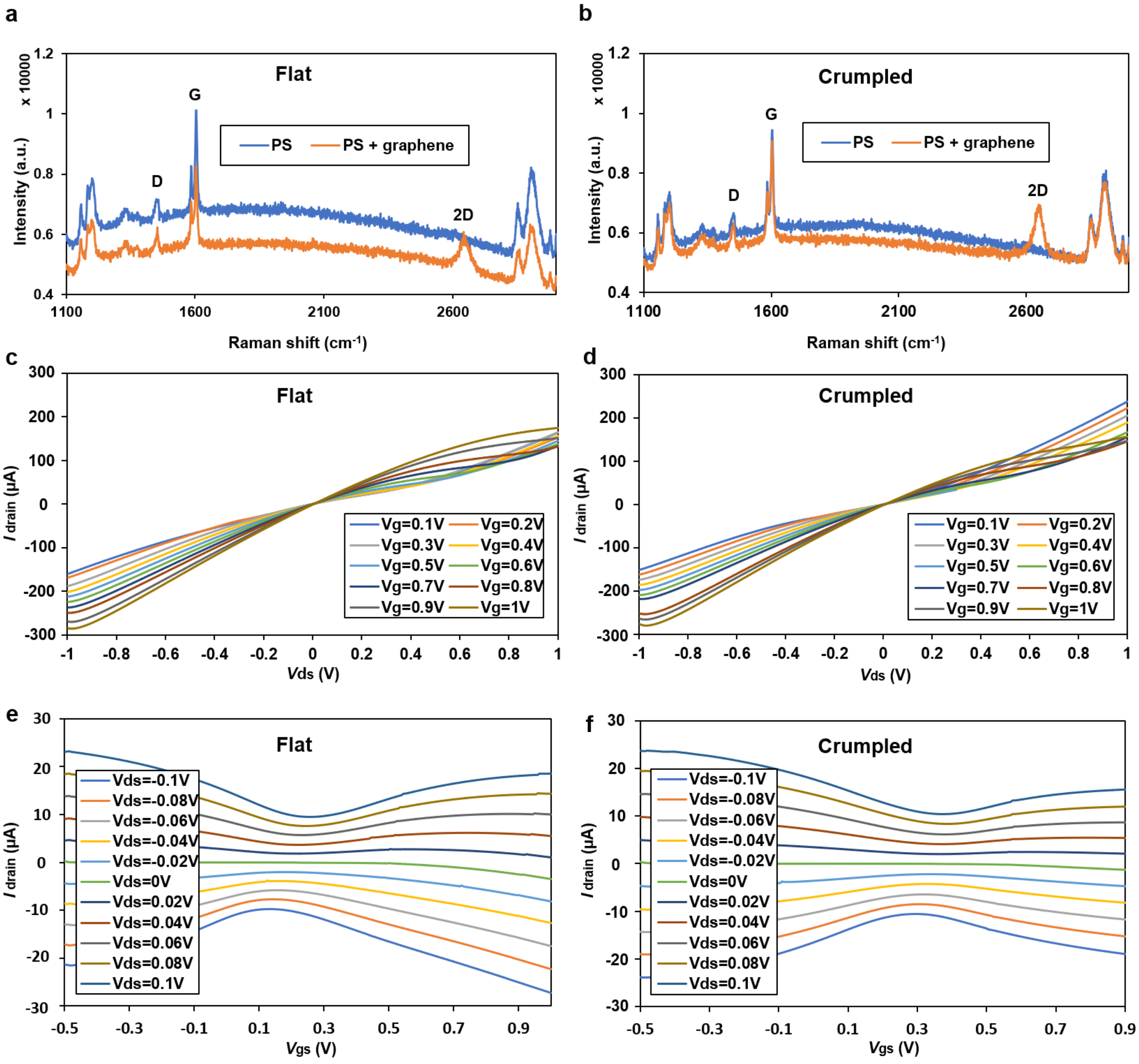

Supplementary Figure 3. Characterization of flat and crumpled graphene FET. **a**, **b**, The Raman D-to-G peak of the flat and crumpled graphene had similar intensity ratio. The background Raman spectrum of PS substrate is shown. **c-f**, Charge transfer characteristics of the fabricated FET using fluid gate. **c**, **d**, Both V_{ds} versus I_{ds} with the variation of V_g showed slightly curved relationship due to Dirac point. **e**, **f**, V_g versus I_{ds} with the variation of V_{ds} graphs also showed shift in the Dirac point.

Supplementary Note 1.

Raman spectroscopy analysis on VHS substrate

As shown in the Fig. 1d and Supplementary Fig. 3a-b, we had Raman spectroscopy. However, as polystyrene (PS) substrate has Raman peak close to graphene G-peak, so the Raman peak marked as “G” in the plot is actually ‘graphene G peak + one of PS’. To observe the G peak without the peak from PS, we prepared flat and crumpled samples on VHB substrate (because VHB does not have Raman peak close to graphene G peak). The following is the sample preparation protocol;

1. Transfer graphene on a PDMS stamp
2. Transfer graphene onto VHB substrate.
    a. For Flat sample: (VHB tape was put on a slide glass because the soft VHB nature made hard the contact printing process.)
    b. For crumpled sample: 100% prestrain was applied in *x*- and *y*-axes to apply same amount of prestrain as crumpled graphene on PS substrate.

The results are below;

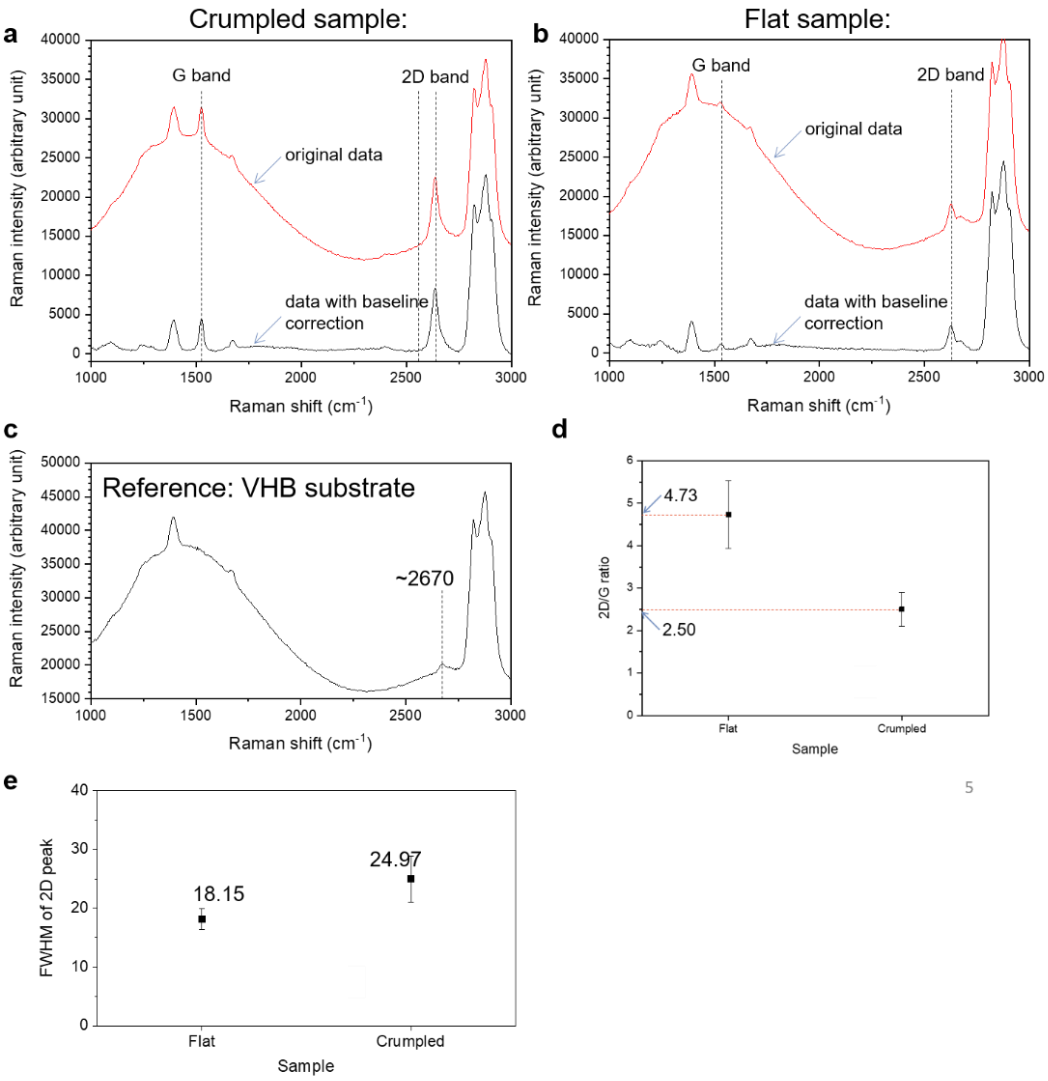

Supplementary Figure 4. Raman spectroscopy results. a, crumpled sample. **b,** flat sample. **c,** substrate only. **d,** 2D/G peak ratio. **e,** FWHM of 2D peak.

Based on 2D/G intensity ratio (d), both crumpled and flat samples showed larger than 2. FWHM of 2D peak was analyzed, and in both samples, FWHM of 2D peak indicates monolayer graphene. (For monolayer graphene, FWHM < 30cm^{-1}, *Nano Lett.* 2007, 7, 2, 238-242). VHB has a Raman peak at ~2670 cm-1 and in crumpled sample, and it was overlapped with graphene's 2D peak. That would have made crumpled graphene sample's FWHM value larger than flat sample. 2D peak center was also analyzed to be larger from crumpled sample (2636.4 cm-1 vs. 2628.2 cm-1) with similar reason. . Based on our Raman spectroscopy analysis, both crumpled and flat graphene samples were monolayer (potentially with small number of bilayer islands due to the graphene growth process, CVD). In addition, since crumpled graphene is three-dimensional structure, out-of-plane structures may interact or even touch each other, but spectral analysis does not indicate apparent evidence of local folding/touching/ interacting of graphene structures. We have added this result in the supplementary Fig. 3. Also there is a previous report that the graphene remained monolayer after the same crumpling process (*Adv. Mater.*, 28: 4639-4645). (*n*=5)

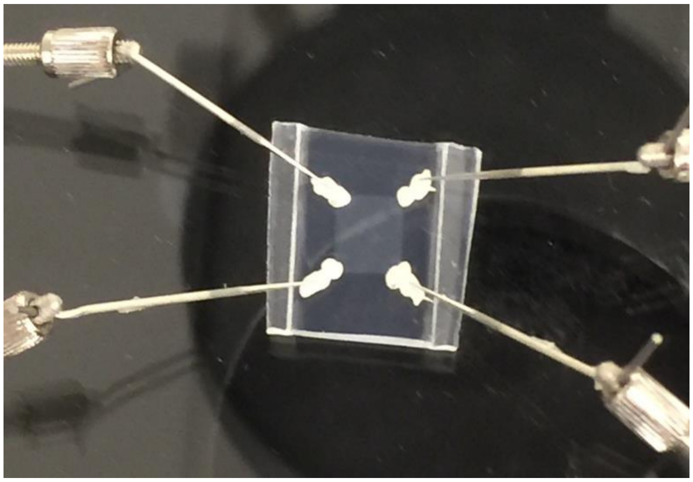

Supplementary Figure 5. Sheet resistance measurement by Van der Pauw method.

Supplementary Table 1. Four-point measurement data.

	Constant Current<br>(100 μA, 1 to 2 and 1 to 3))
Measured Voltage 1 to 2	8~10 mV
Measured Voltage 1 to 3	8~10 mV

Using the equation from reference paper (*Chin. Phys. B* Vol.26, No.6 (2017) 066801). The sheet resistance is ~450 Ω, which is in concordance with known values.

Supplementary Note 2.

Note for Dirac voltage point determination.

The measurements were repeated 6 times every two minutes for each data point (each concentration of target nucleic acids) and the Dirac point was confirmed to be stabilized when at least the last two measurements of Dirac points were same. The device was rinsed with fresh PBS every 3 measurements. Once the device and the Dirac point became stable, the Dirac points were same over many repeats of measurements. Supplementary Fig. 4 shows stable Dirac points for 10 repeats of measurements over 96 min. Also, note that negative control tests showed that the signals were much smaller or negligible compared to the signal generated by the target nucleic acids (Fig. 2f, 2h and 2i).

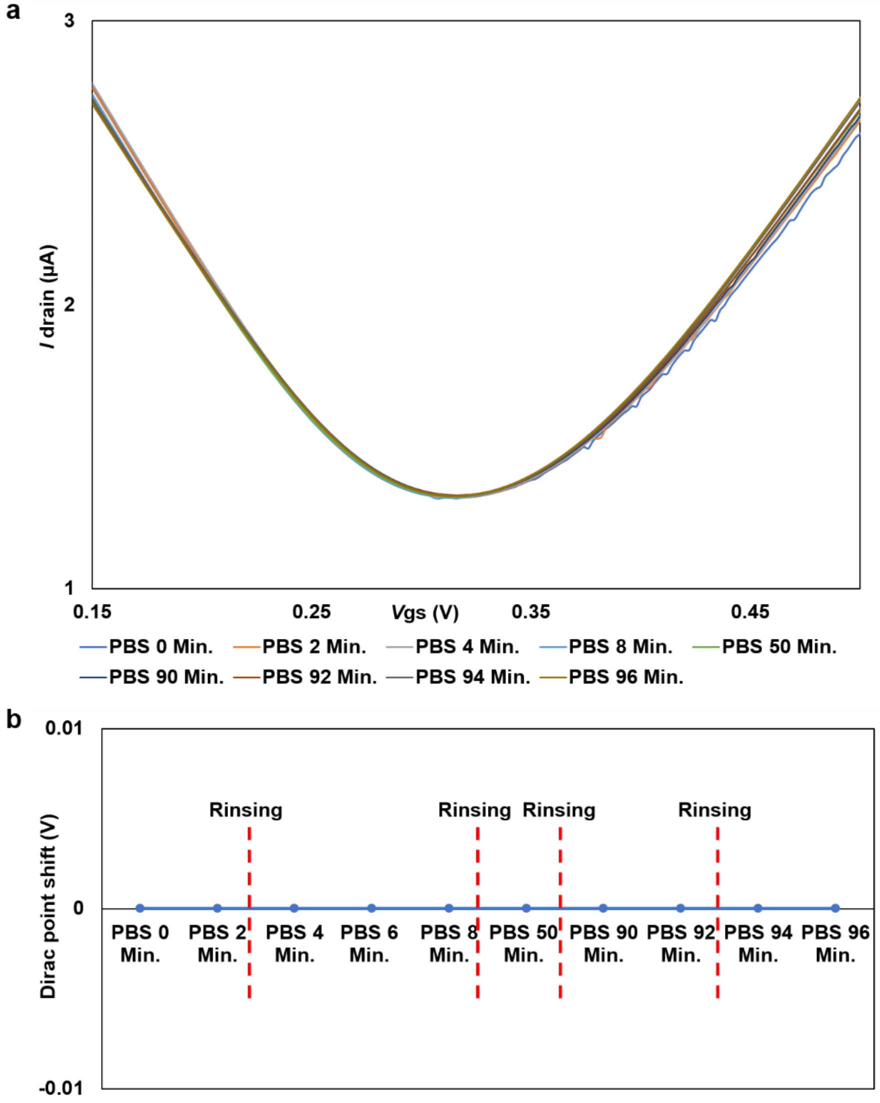

Supplementary Figure 6. Dirac voltage point stability test. The *I-V* measurements were repeated over time to evaluate the Dirac voltage point (the gate voltage at the minimum drain current point) was stable. **a,** The *I-V* curve of the probe functionalized device was measured in 1×PBS at the given time in the legend. **b,** The device was rinsed with fresh PBS buffer solution at points marked with red dot line. The Dirac voltage values were stable and did not showed shift. The increment of the gate voltage was 2 mV.

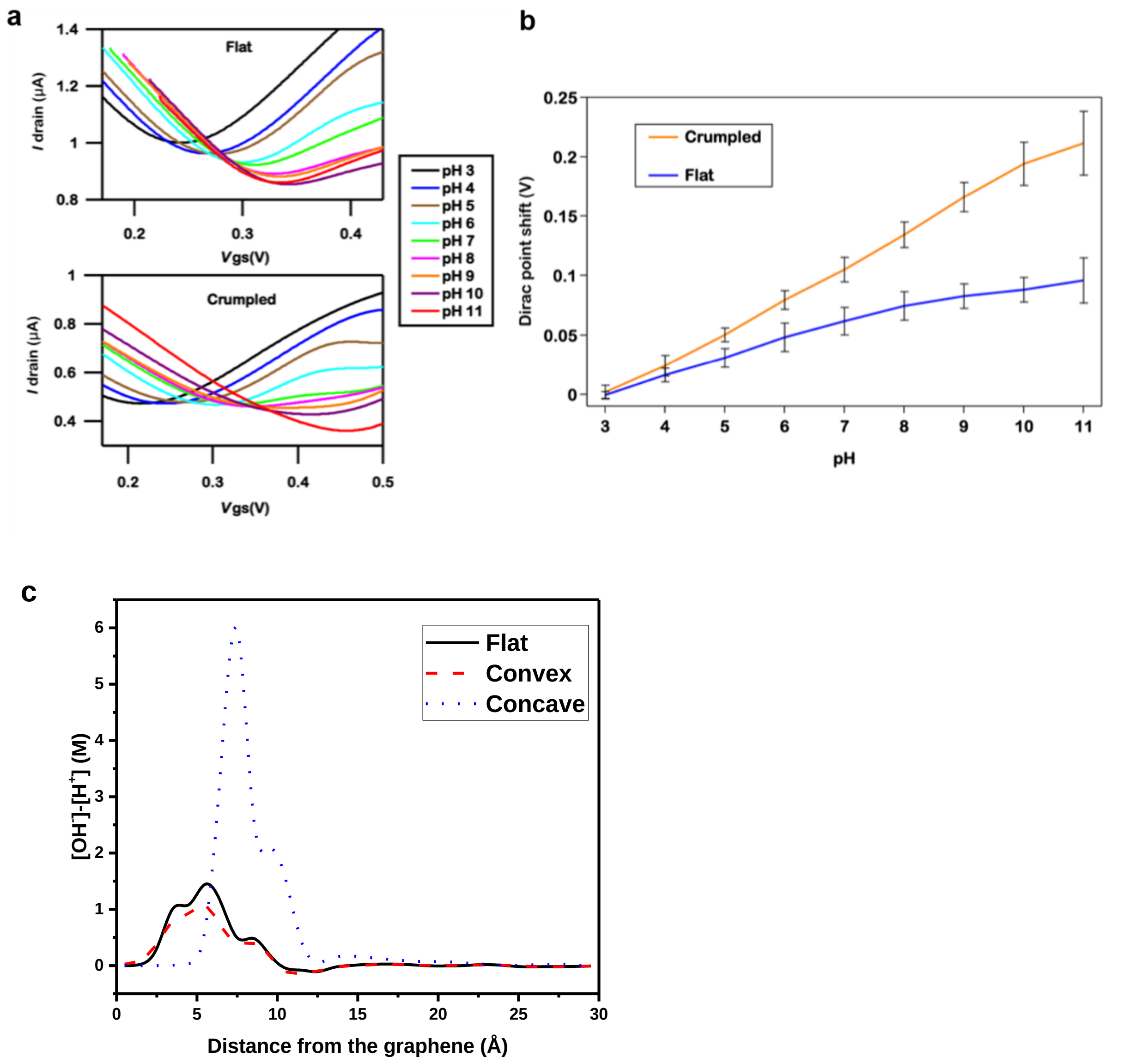

Supplementary Figure 7. pH responses of flat and crumpled FETs. The measurements were performed in a PBS buffer from pH 3 up to 11 at constant drain-source voltage of 0.05V. The pH of PBS was adjusted by adding hydrochloric acid (HCl) or sodium hydroxide (NaOH). **a,** *I-V* relationship of the flat (top) and crumpled (bottom) graphene FET sensors at different pH values. **b,** Dirac point shifts of the FET sensor plotted as a function of pH values. The average values of Dirac point shift with pH were 12.2 mV/pH for flat FET and 27.2 mV/pH for crumpled FET. *n*=5, mean ± std. **c,** The molar concentration difference of OH^- and H^+ as a function of the distance from the positively charged graphene surface for flat, convex and concave cases. A much higher negative charge concentration is observed for the concave regions. This higher charge concentration induces a stronger change in charge carrier density of graphene resulting in a larger Dirac point shift in experiments.

Supplementary Table 2. DNA, PNA and RNA sequences used in the experiments

	Sequence
22-mer (Probe)	NH2-AACCACACAACCTACTACCTCA-3’ (DNA)<br>5’-AACCACACAACCTACTACCTCA-OO (PNA)
Let-7b (Target)	5'-TGAGGTAGTAGGTTGTGTGGTT-3’ (DNA)<br>5'-UGAGGUAGUAGGUUGUGUGGUU-3’ (RNA)
miR-21 (Negative Control)	5'-TAGCTTATCAGACTGATGTTGA-3' (DNA)<br>5'-UAGCUUAUCAGACUGAUGUUGA-3' (RNA)
AFM imaging 1	5’-TGA AAG IGT TTT AAT AAT AGA ATT TTA AAA IAC TIG TAI A-3’
AFM imaging 2	NH2-CCT TAT TTC TAC CAG TCT TTT AAA ATT CTA TTA TTA AAA CCC TTT CA-3’
3 nt short target for charge layer distance experiment	5'-TGAGGTAGTAGGTTGTGTG-3’

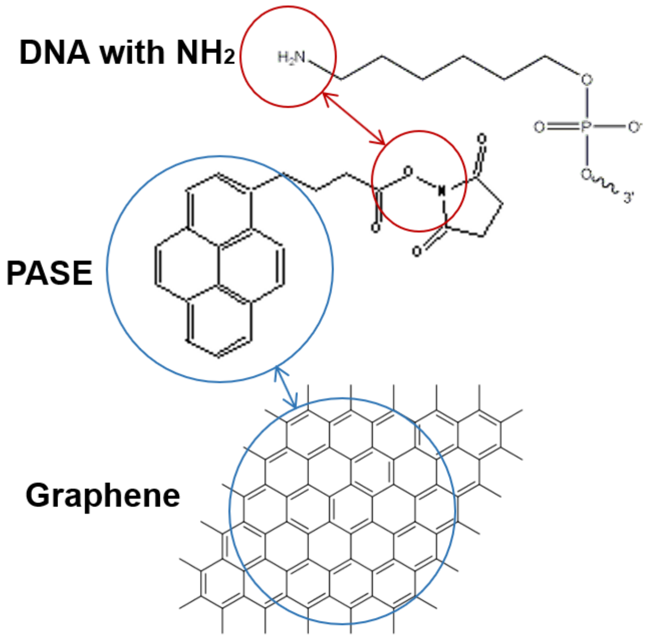

Supplementary Figure 8. Scheme of probe DNA immobilization process. Probe DNA has a primary amino group positioned at the 5’-end with a standard (C6) spacer arm (top). The amino group is covalently reacted with one side of PASE molecules (middle). The other side of PASE is π- π stacked with graphene (bottom).

Supplementary Note 3.

Sips model fitting results.

In Fig. 2g, the absolute value of the Dirac point shift dataset has been plotted as circular solid markers (red for crumpled and blue for flat). The Sips fitting lines are shown as red and blue solid lines. A red dotted line is the detection baseline that is estimated from the average value of the negative binding measurements shown in the main dataset figure.

The Sips model (*J Chem Phys* **16,** 490 (1948), *ACS Nano* **10,** 8700 (2016)) is adapted to fit the DNA hybridization specifically bound on the flat and crumpled graphene surface. Single stranded DNA target molecules, Let-7b, are specifically bound on the other complimentary single stranded DNA, 22-mer probe, molecules. They form duplex DNA molecules. The Sips model is the best fit to describe the relation of the shifted Dirac point voltage responding to the absorbed DNA concentration on the crumpled graphene surface to the target concentration (C) dissolved in a buffer,

$$|\Delta V_D(V)| = A\frac{(C/K_a)^a}{1+(C/K_a)^a}$$

, where A is the maximum value of Dirac point shift with all probe sites occupied, K_a is the equilibrium dissociation constant, and a is the characteristic parameter of the Gaussian distribution of DNA-binding energies on the graphene surface.

Supplementary Table 3. Fitting Parameters used in Sips model in this work.

	crumpled	flat
A	0.122 ± 0.007 V	0.072 ± 0.002 V
a	0.200 ± 0.021	0.436 ± 0.053
K_a	1.12e-11 ± 9.44e-12 M	9.71e-11 ± 3.37e-11 M

The Sips model is commonly applied to describe a statistical distribution of the molecular (or gas) adsorption energies on a solid surface especially when the adsorption energies of the binding sites are heterogeneous other than homogeneous.

The A value corresponds to the saturation value of Dirac point voltage when all probe molecules are fully occupied. The saturation voltage for the crumpled graphene device is about 0.122 V which is about 1.7 times larger than 0.072 V for flat. The saturation voltage becomes larger when the graphene is crumpled, in a good agreement with the capacitance simulation results. i.e., the voltage is inversely proportional to the capacitance and the capacitance of the crumpled is reduced in a half of the value of the flat.

The association constant a characterizes the energy distribution of the DNA adsorption isotherm on the surface. The a value is in a range from 0 to 1. When a =1, the Equation turns into the Langmuir adsorption isotherm, in which all the DNA-binding sites have the same binding energy. If a decreases, the distribution curve shows a transition from a steep slope to a low slope as the target concentration increases. For the limit of a = 0, it results in a constant value. From the crumpled graphene results, a = 0.2 reflects the broader DNA-binding energy distribution on the crumpled as compared with 0.436 for

the flat case. It assumes that the broad energy distribution must be correlated with heterogeneous DNA-binding sites, such as the deep valleys, the slopes, or the peaks of the crumpled surface.

The dissociation constant K_a is a strong relationship with the binding DNA length. The K_a value decreases exponentially as the adsorbed molecular size increases (ref. ACS Nano 2016 10 8700). From the data set, two K_a values are very similar between the crumpled and flat devices.

As shown in the figure, the detection limit is actually shifted from a femto-Molar for the flat down to an atto-Molar for the crumpled. The Sips fitting parameters A and a describe, with a statistical method, that the crumpling effects enhance the capacitance and also lower the detection responding slope so that the crumpled-graphene system can detect the adsorbed DNA molecules in an atto-molar scale concentration.

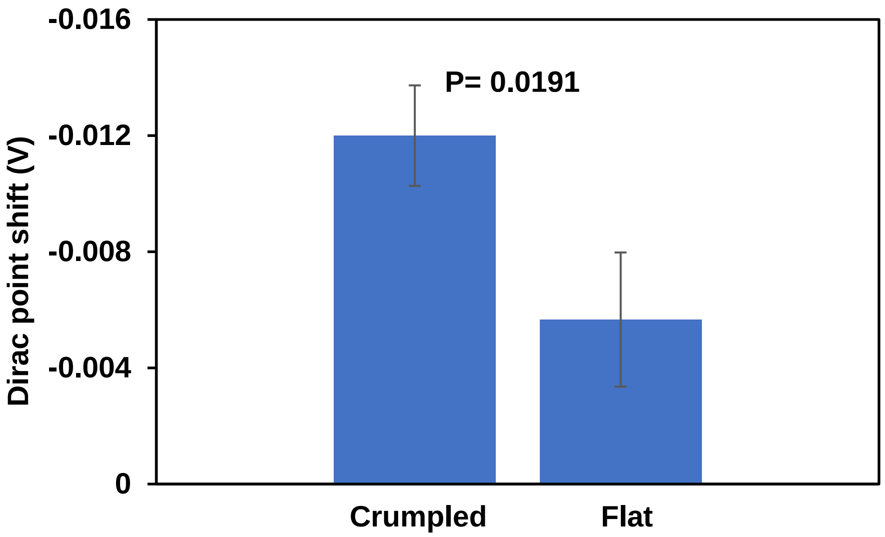

Supplementary Figure 9. P-value of Dirac point shifts between crumpled and flat graphene FET biosensor at 20 aM of target DNA hybridization. The graph shows clear difference between two data points with p-value of 0.0135.

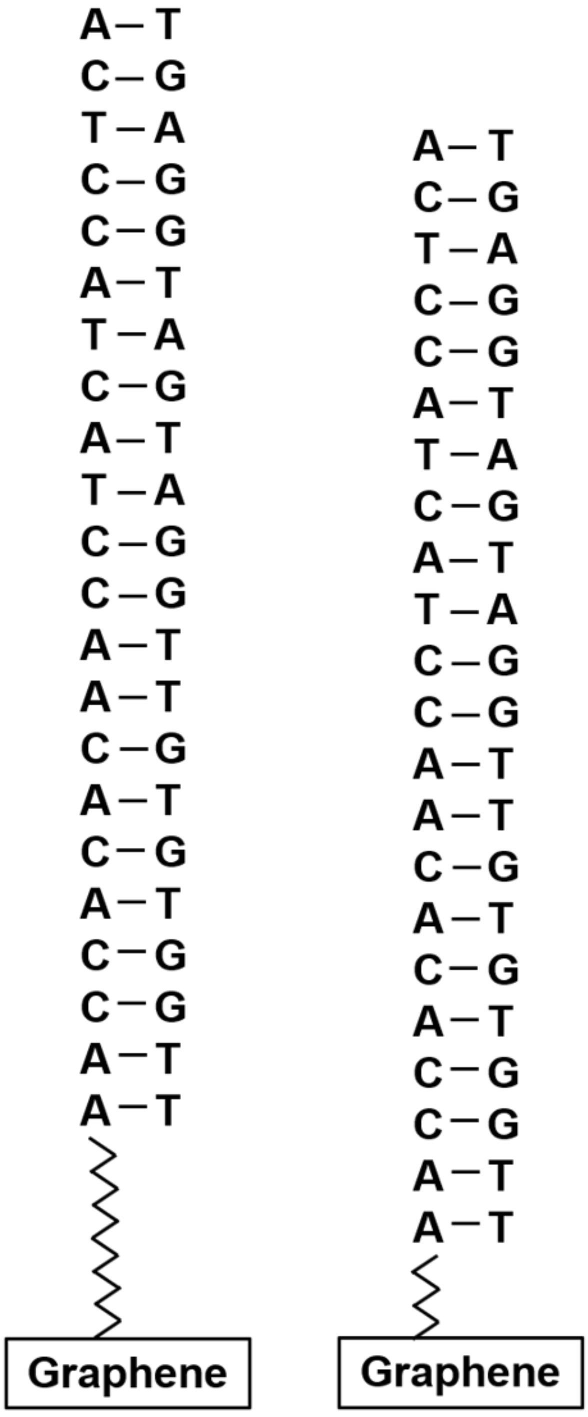

Supplementary Figure 10. Schematic of the molecule with the DNA and PNA sequence and linker molecule structure. **Left**, the structure that was used for the DNA/DNA hybridization experiments. **Right**, the structure that was used for the PNA/DNA hybridization experiments. PNA has 7 carbon shorter distance between nucleic acids and graphene surface.

Supplementary Note 4.

Consideration of convection-diffusion-reaction model.

Taking into account convection-diffusion-reaction considerations, evaporation induced convection and surface roughness effect on molecular absorption may facilitate the transport of nucleic acids to the graphene surface, reducing the diffusion-reaction time significantly and contributing to the high-sensitivity detection. Also, the surface roughness of crumpled graphene may influence the molecular reaction process, compared to a flat graphene.

In general, while a small volume of water droplet is placed on a solid substrate at a room temperature, the water droplet evaporates and drives convention flow. The convection flows down from the top surface of the droplet to its bottom solid-water surface and then flows up from the edge to the top. The convection flow rates depend on temperature and humidity. Due to convective flow, the molecules at the central region move along downstream to the bottom surface and spread over the surface in a radial direction. The flow speed varies inside the droplet. The speed is highest at the top central region and becomes much slower on the bottom region (where diffusion wins). The typical value ranges from 0.1 to 100 um/s at room temperature (*J. Phys. Chem. B* **118,** 2414-2421 (2014)). In other words, the molecules approach to the surface fast at the central region and slow down on the surface where diffusive transport is dominant (*Drying Technology* **37,** 129-138 (2019)).

Considering that target molecules are across r = 3mm, radius of water droplet, the diffusion time is proportional to r/2D, D is diffusion constant, and convective time is proportional to the evaporation rate, Q, is about 0.33 μl/min inside the probe station. Peclet number, Pe, can characterize the mass transport as diffusion-limited or reaction-limited (*Nature Biotechnology* **26,** 417–426 (2008)). For convection and diffusion (D = 100 $\mu m^2/s$) (*ELECTROPHORESIS* **23,** 2794-2803 (2002)), If Pe = diffusive time/convective time is >> 1, reaction limit dominates the system while if Pe<<1, diffusion limit dominates In our case, Pe is ~10, which means that chemical reaction occurs slowly while target molecules are supplied relatively fast to the surface. In the reaction-limited, the binding reaction is the major time obstacle to collect all target molecules on the sensor area.

By applying Langmuir kinetics to assume target bindings on the reactive surface, the target concentration can be estimated by:

$$\frac{\partial[TP(t)]}{\partial t} = k_{on}[T](P_m - [TP(t)]) - k_{off}[TP(t)]$$

where P_m is the probe concentration, *[T]* target concentration, *[TP]* target-probe complex concentration, k_{on} is an associate rate constant, k_{off} is a dissociation rate constant. For chemical reactions when all molecules can diffuse to the surface, the equation above can be solved:

$$[TP(t)] = P_m \frac{[T]/K_D}{1 + [T]/K_D}\left(1 - e^{-(k_{on}[T] + k_{off})t}\right)$$

where $K_D = k_{off}/k_{on}$ is the equilibrium dissociation constant. Given parameters, K_D = 10pM (from Sip's fitting results), $P_m = 1\text{x}10^3/\text{um}^2$ (*ACS Nano* **10,** 8700 (2016)), k_{on} is 10^6 M^{-1} s^{-1}, and $K_{off} = 10^{-5}s^{-1}$, as shown in the plot of *[TP(t)]* vs time, the equilibrium time reaches to ~10^5 seconds (16 hours) for sub pico-molar target concentration. Since the equilibrium time decreases as the concentration increases, the graphene sensor is limited to pico-molar sensitivity within one-hour equilibrium state (*Nano Lett.* **5,** 803 (2005)).

Before reaching the equilibrium state, the one-hour practical incubation time indicated by arrow, about 16.5 % of target DNA concentration molecules can be bound on the surface. Graphene sensors are experimentally able to detect them (*Nano let.* **18,** 3509 (2018)).

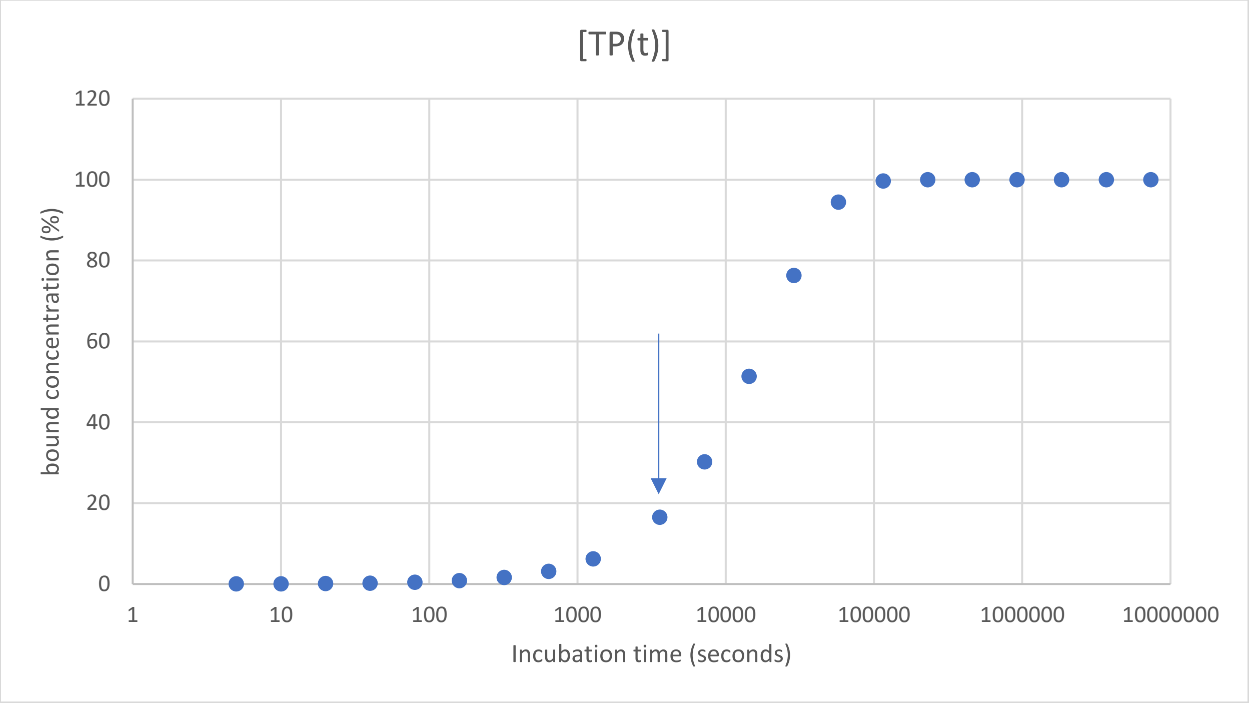

Supplementary Figure 11. The rate of DNA molecules bound on the graphene surface over time. The arrow indicates about 16.5 % of target DNA concentration molecules binding on the surface after 1 hour of incubation.

The surface roughness of crumpled graphene may also influence the molecular adsorption process, compared to a flat graphene. Crumpled graphene forms randomly oriented valleys-and-peaks surface. The RMS roughness is about 500nm between valleys and 300 nm for their depth (*Nano Lett.* **15,** 7684–7690 (2015)). When the molecular size is larger than 500nm, conformational entropic trap holds the molecules inside the valley. On the other hand, when the size is much smaller, the molecules move “relatively” freely inside and outside of the rough surface. The molecules face increased interactions, such as Van der Waals, electrostatic force, and hydrophobicity. Thus, they stay in the valleys for longer than typical diffusion time scale (*Nano Lett.* **18,** 3773-3779 (2018)). Ruggeri et al, claimed the well depth of 330 nm gives up to 5 k_BT configurational free energy barrier, W, and the molecular residence time is proportional to $\exp(W/k_BT)$. The molecules stay longer by 10^2 X of diffusion time scale. Practically, molecular adsorption rate is increased with increasing surface roughness (*Langmuir* **22,** 10885-10888 (2006)).

Therefore, collectively evaporation induced convection and surface roughness effect on molecular absorption possibly facilitate the transportation of nucleic acids to the graphene surface and may reduce the diffusion-reaction time significantly and contributed to the high-sensitivity detection.

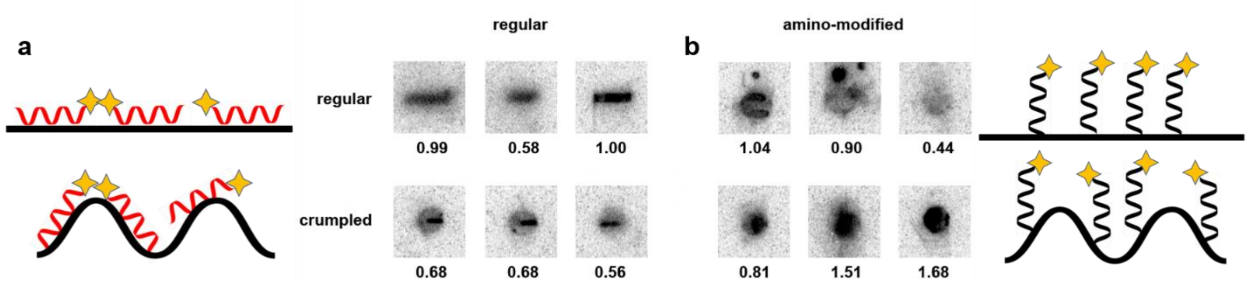

Supplementary Figure 12. Quantification of DNA using radioactive labeling. Radioactive isotope phosphorus 32 (P32, yellow star) was labeled at the end of target (red) and probe (black) DNA. **a**, target DNA was absorbed on both flat and crumpled graphene by π-π stacking and quantified. **b**, probe DNA was immobilized on both flat and crumpled graphene. All the experiment conditions were same with Fig. 2. The number represent the relative intensity normalized with respect to the third image (1.00). The results show that density of DNA on the flat and the crumpled graphene is in the same order.

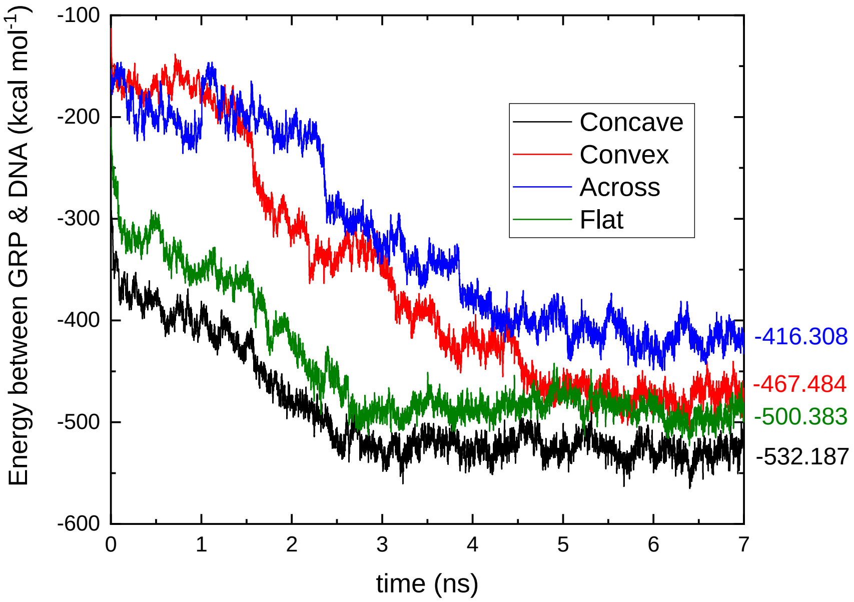

Supplementary Figure 13. The interaction energy between graphene and DNA strand as a function of simulation time for the different configurations of DNA on the crumpled graphene as well as on flat graphene.

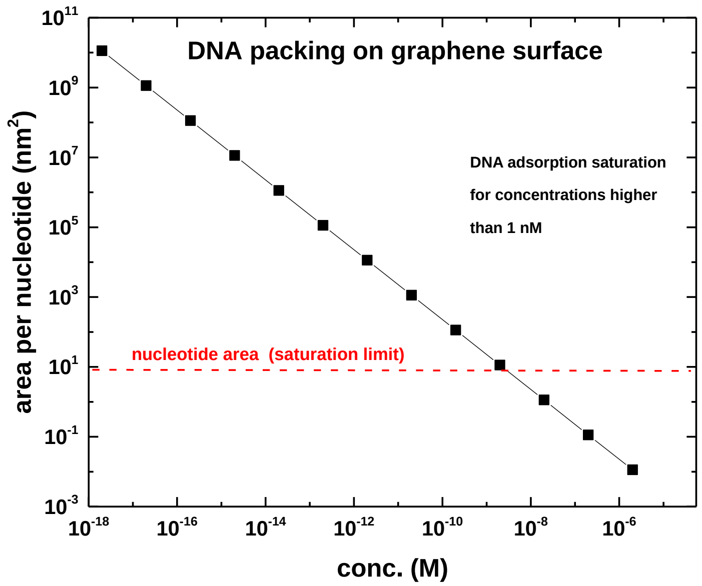

Supplementary Figure 14. The packing of DNA molecules adsorbed onto the graphene surface. The area per nucleotide is plotted along the y-axis if the entire DNA concentration were to be adsorbed as a single layer on the graphene surface. The area occupied by one DNA nucleotide is assumed to be ~8 nm^2 based on DNA nucleotide size (the dashed line). Concentrations higher than 10^{-9} M results in extreme packing (area per nucleotide smaller than nucleotide size) which indicates saturation of a monolayer of DNA molecules on the graphene surface.

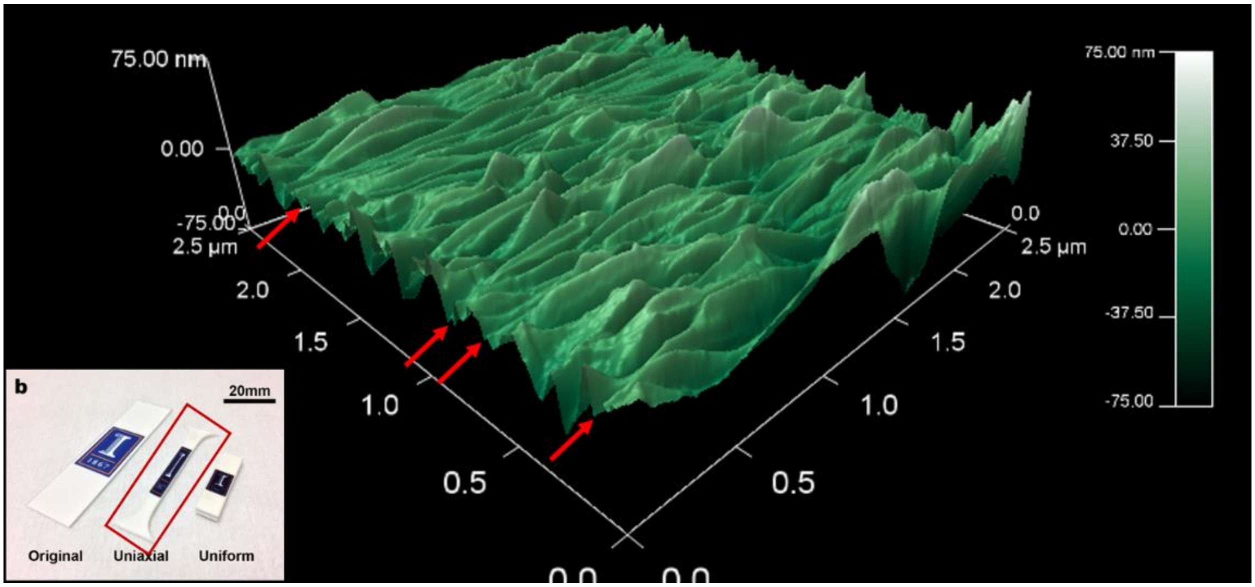

Supplementary Figure 15. AFM image of uniaxially crumpled graphene. The red arrows indicate fine crumples with a few nanometer sizes.

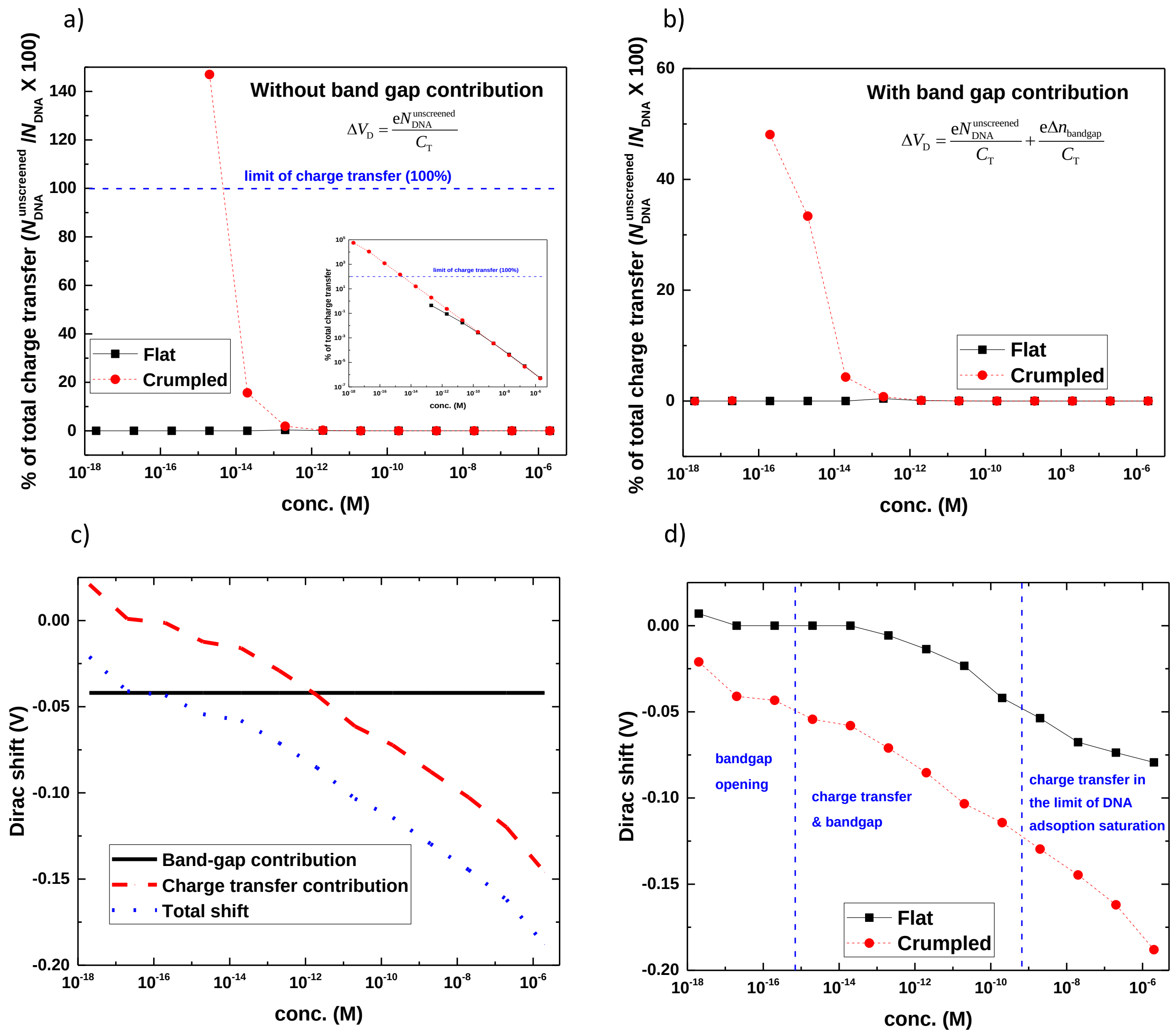

Supplementary Figure 16. Percentage of DNA charge transfer to graphene to match the experimental Dirac point shift **a,** without band gap contribution, and **b,** with band gap contribution. Without band gap, the required charge transfer exceeds the maximum available limit indicating that charge transfer is not the only mechanism by which Dirac point shift occurs. **c,** Contribution of charge transfer and band gap (a constant Dirac shift due to band gap is assumed) to the total Dirac point shift. **d,** Three different regions are defined. < 200 aM region where the band gap opening is dominant, >200 aM and <1nM region where the charge transfer becomes significant in addition to the band gap opening and >1nM region where the charge transfer is dominant while DNA adsorption saturation on graphene takes place.

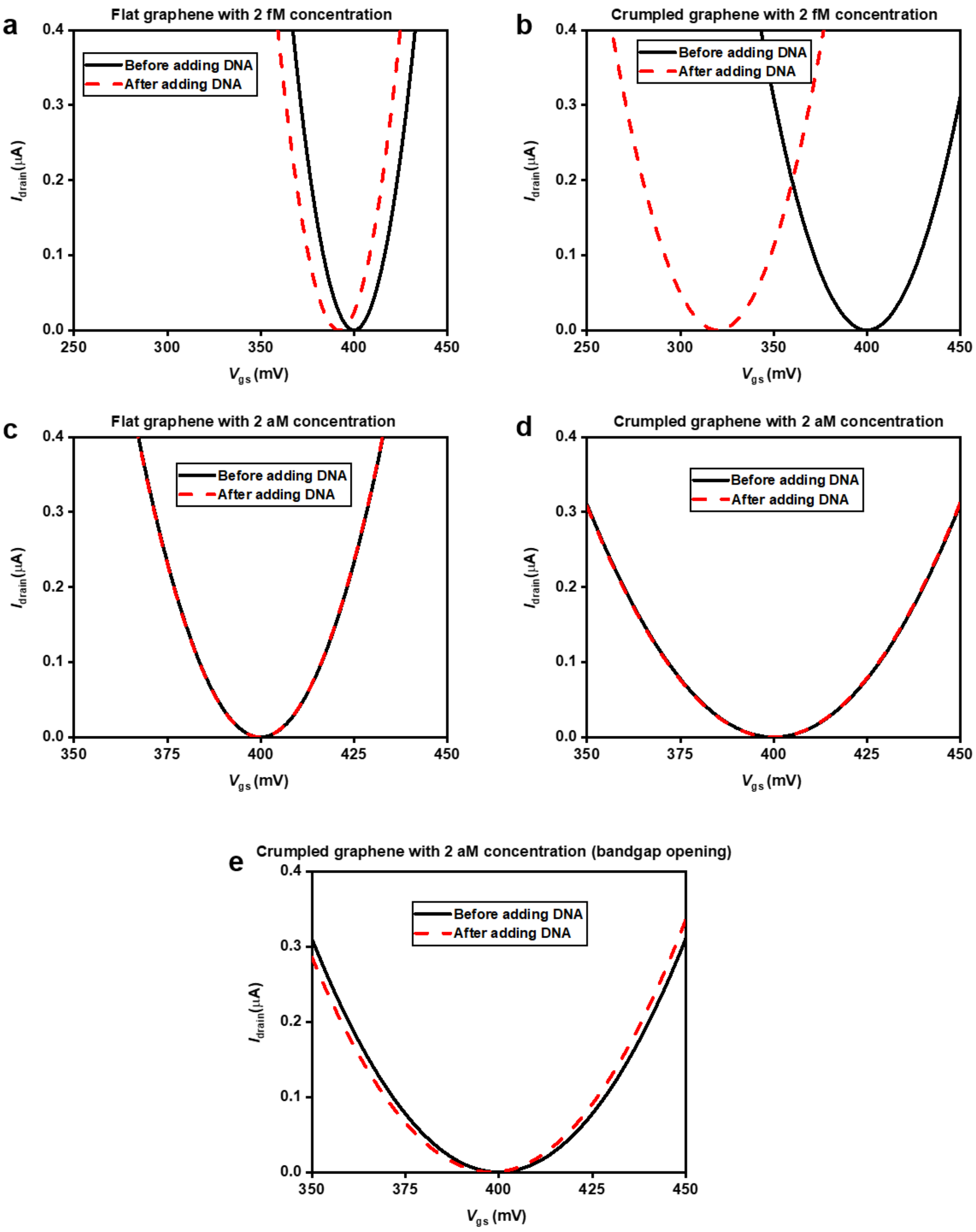

Supplementary Figure 17. The modelled I-V curves before and after adding 2fM complementary DNA for **a**, flat and **b**, crumpled graphene. The I-V curves before and after adding 2aM complementary DNA for **c.** flat and **d**, crumpled graphene. The potential shift in **a**-**d** is computed assuming 80% (for flat graphene) and 10% (for crumpled graphene) ionic screening using $\Delta V_{\mathrm{D}} = \frac{\mathrm{e}N_{\mathrm{DNA}}^{\mathrm{unscreened}}}{C_{\mathrm{T}}}$. The Dirac point shift due to a bandgap opening in 10^{-7}% of the crumpled graphene after adding 2aM complementary DNA is ~5mV and the corresponding I-V curve is plotted in **e**.

Supplementary Table 4. The calculated band gap (in eV) of graphene in the presence of single DNA bases with three different orientations using DFT and GW. The GW values are reported in parenthesis. The crumpled graphene has a wavelength of 0.818 nm and an amplitude of 0.26 nm. See Supplementary Fig. 17 & 18 for the schematic of the orientations.

Case		Flat graphene	Crumpled zigzag graphene	Crumpled armchair graphene
No DNA		0 (GW: 0)	Metal (GW: metal)	0.1325 (GW: 0.4224)
A 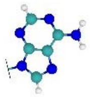	Orientation I	0.0046 (GW: 0.8535)		0.1434 (GW: 1.7641)
	Orientation II	0	Metal	0.1430 (GW: 0.5508)
	Orientation III	0		0.1491
C 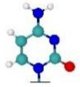	Orientation I	0.0053 (GW: 0.8568)		0.1418 (GW: 1.7493)
	Orientation II	0	Metal	0.1434
	Orientation III	0		0.1467
G 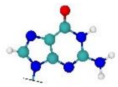	Orientation I	0.0049 (GW: 0.8518)		0.1522 (GW: 1.7504)
	Orientation II	0	Metal	0.1481
	Orientation III	0		0.1305
T 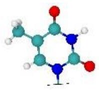	Orientation I	0.0054 (GW: 0.8562)		0.1485 (GW: 1.7477)
	Orientation II	0	Metal	0.1474
	Orientation III	0		0.1448

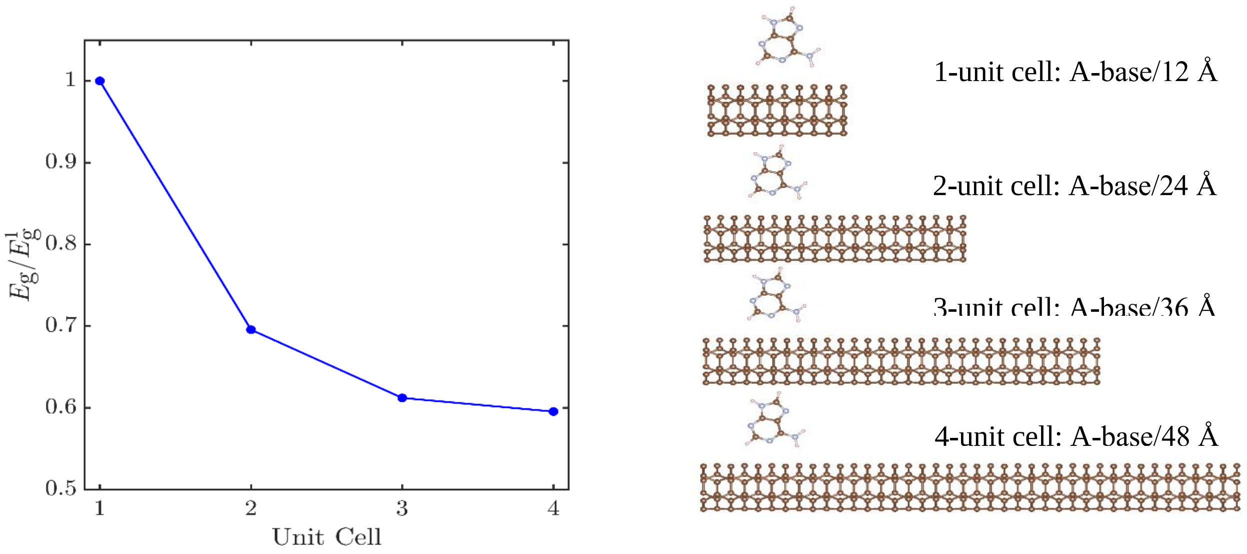

Supplementary Figure 18. Bandgap as a function of the size of graphene unit cell for a single DNA base. The bandgap is normalized by the bandgap of 1-unit cell. The width of all the cells is ~12 Å and only the length is varied. The graphene bandgap in 4-unit cell is about 60% of that of 1-unit cell which is still a significant bandgap value. This shows that the effect of a single DNA base on the bandgap of graphene is long range.

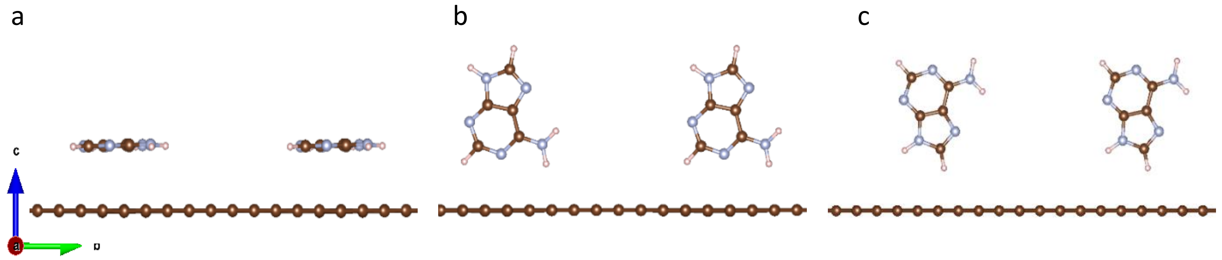

Supplementary Figure 19. The nucleobase orientations above the pristine graphene surface used in the DFT/GW simulations. a) orientation 1, where the plane of the base ring is parallel to the a-b plane, b) orientation 2, where the plane of the base ring is perpendicular to the a-b plane (or parallel to the a-c plane), c) orientation 3 is similar to orientation 2 where the ring is rotated 180° around the a-axis.

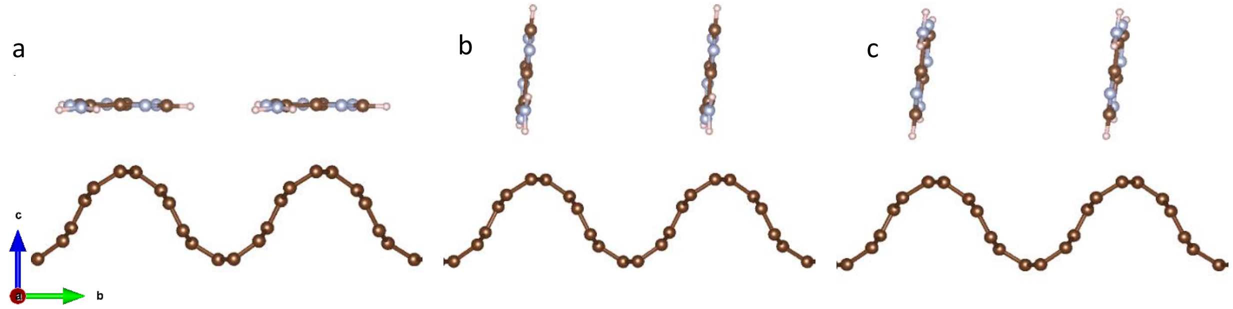

Supplementary Figure 20. The nucleobase orientations above the crumpled graphene surface used in the DFT/GW simulations. a) orientation 1, where the plane of the base ring is parallel to the a-b plane, b) orientation 2, where the plane of the base ring is perpendicular to the a-b plane (or parallel to the a-c plane), c) orientation 3 is similar to orientation 2 where the ring is rotated 360° around the a-axis.

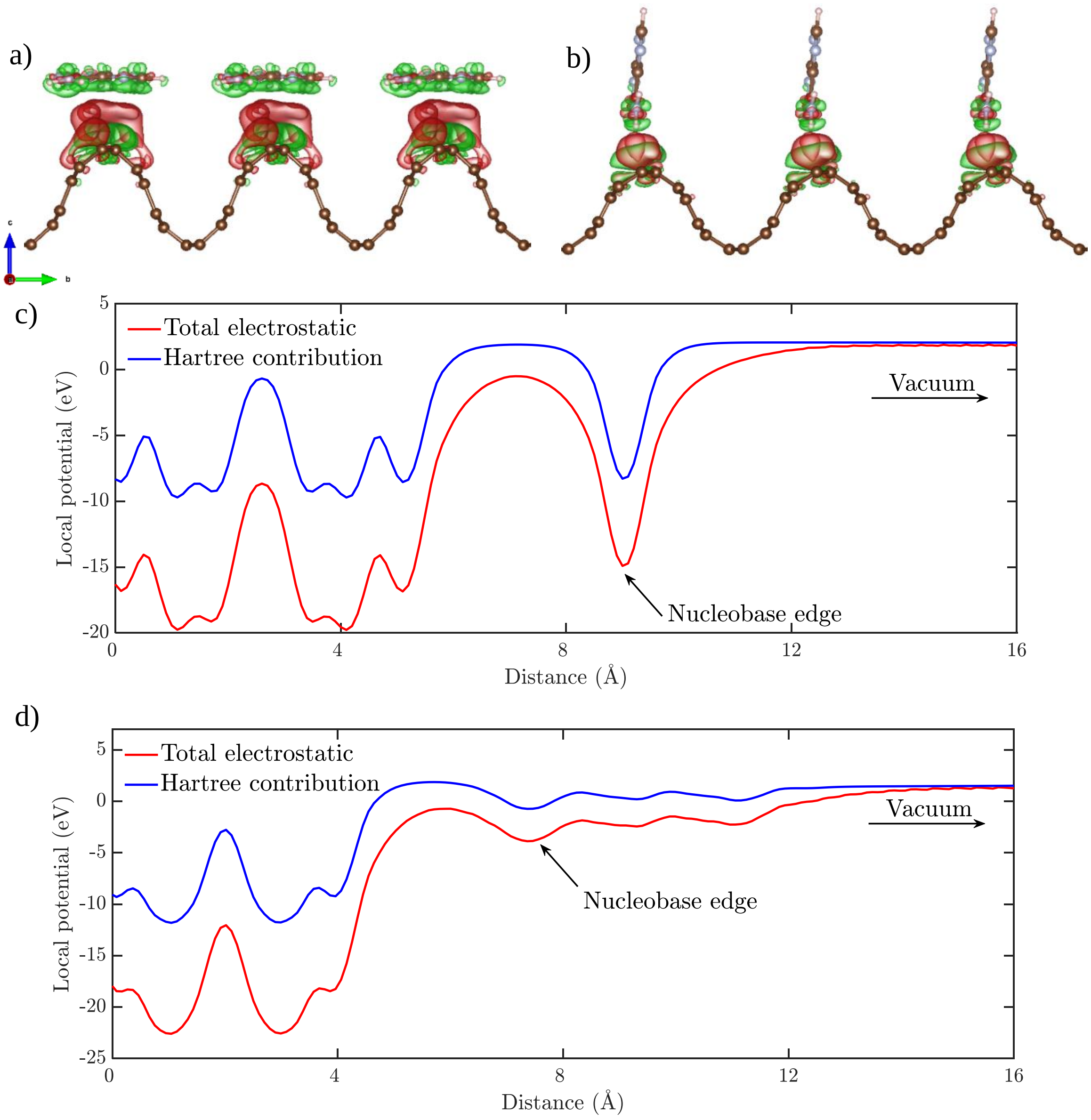

Supplementary Figure 21. Interaction of the nucleobase with the crumpled graphene surface for different orientations: a) interfacial charge density for parallel orientation (orientation 1) and b) interfacial charge density for perpendicular orientation (orientation 2) (green and red colors represent negative and positive charge difference, respectively). Local potential versus the distance from the bottom of the crumpled graphene, c) for orientation 1 and d) for orientation 2 along the *c*-direction (red curve is the total electrostatic potential and blue curve is the Hartree contribution).

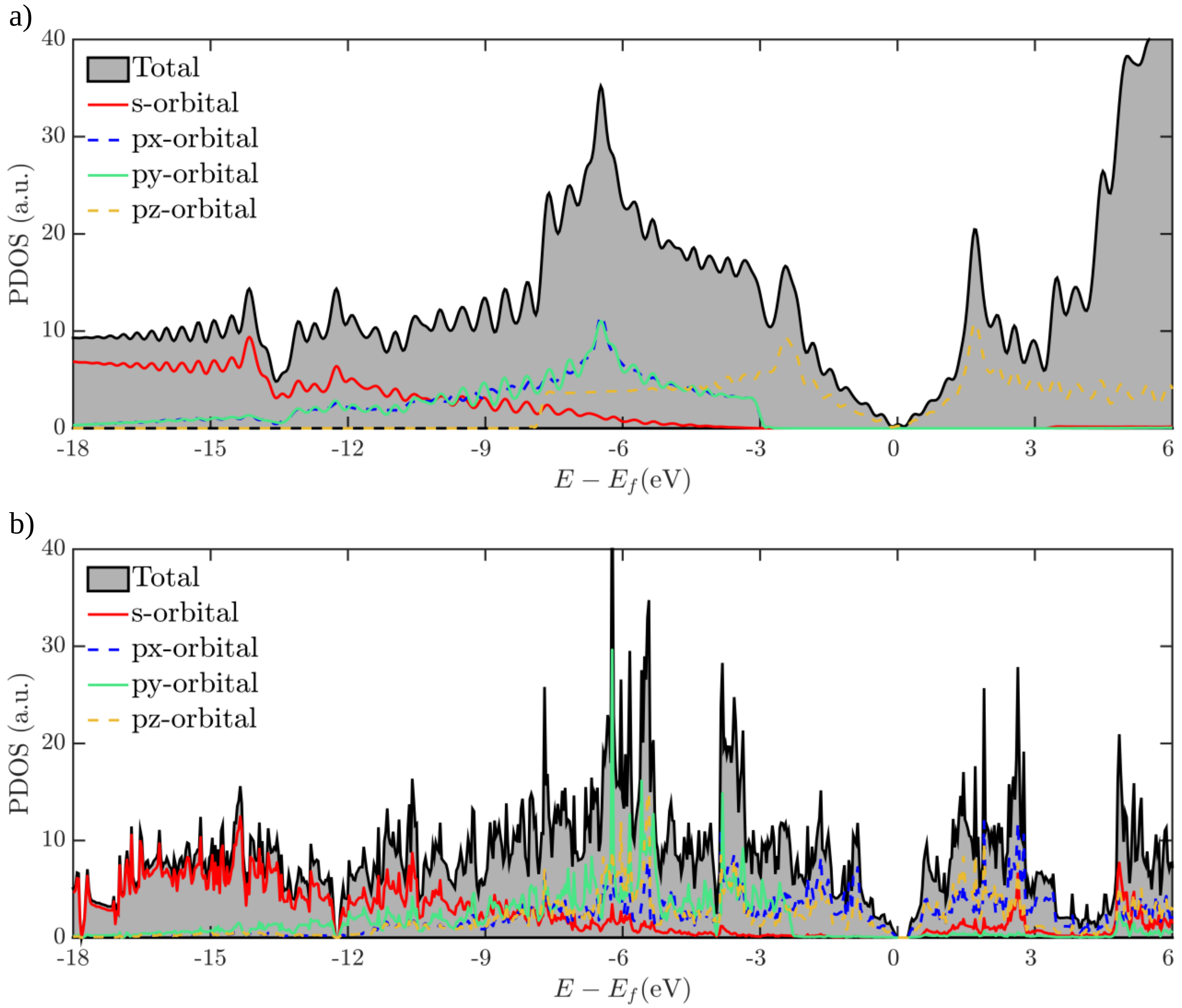

Supplementary Figure 22. Partial density of states for: **a,** pristine graphene and **b,** crumpled graphene for different molecular orbitals. Total density of states (gray filled), s-orbital contribution (red), px-orbital contribution (blue), py-orbital contribution (green), and pz-orbital contribution (orange) are shown.

Supplementary Note 4.

DNA adsorption to the graphene surface

The interaction energies between the DNA and crumpled graphene are calculated for different configurations of DNA. The equilibrium energies for the DNA in the concave, convex, across and flat regions are -532.187 kcal mol^{-1}, -467.484 kcal mol^{-1}, -416.308 kcal mol^{-1} and -500.383 kcal mol^{-1}, respectively. Supplementary Fig. 11 shows the evolution of the interaction energies as the DNA strand binds onto the graphene surface.

We investigated the DNA adsorption onto the graphene by plotting the area per nucleotide (packing) for the concentrations used in this study in Supplementary Fig. 12 assuming all DNA molecules formed a monolayer at the graphene interface. As shown, for the concentrations higher than 1 nM, the area per nucleotide is lower than the area occupied by a DNA nucleotide (based on the size of one nucleotide) indicating an extreme packing if the molecules were hypothetically adsorbed as a monolayer next to graphene. Therefore, DNA adsorption saturation must take place for high concentrations (~1nM and above) and the additional molecules are simply accumulated in layers away from graphene.

Dirac point shift mechanisms for different concentrations

We studied the Dirac point shift for a range of different concentrations to identify the dominant mechanisms by which the shift takes place. First, we excluded the effect of the band gap and calculated the shift solely based on the charge transfer from the unscreened DNA molecules using $\Delta V_{\mathrm{D}} = \frac{\mathrm{e}N_{\mathrm{DNA}}^{\mathrm{unscreened}}}{C_{\mathrm{T}}}$. By matching the experimental ΔV_D, charge transfer ($N_{\mathrm{DNA}}^{\mathrm{unscreened}}$) can be extracted. The ratio of the transferred charge to the total available DNA charge in the solution is plotted for different concentrations in Supplementary Fig. 14a. As shown, for the crumpled graphene, the charge transfer is higher than that of a flat graphene (indicating less screening in crumpled graphene) for the intermediate range of concentrations. In addition, the required charge transfer exceeds the maximum available charge (>100%) for low concentrations indicating that charge transfer by its own is not the only mechanism responsible for the change in the carrier charge density. Next, we assume a constant change in the charge carrier density corresponding to a Dirac point shift of -0.042 V due to the band gap change (we justify the band gap opening in the next section of this supplementary information). In Supplementary Fig. 14b, with the band gap included, the % of charge transfer is estimated by matching the experimental shifts. As shown, for the crumpled graphene, for the two highest concentrations, the charge transfer is almost zero as the band gap is the dominant contributor to the shift. In Supplementary Fig. 14c, we plotted the individual contribution of charge transfer and bandgap (note that due to the complexity of band gap opening, we assume a constant Dirac point shift due to band gap opening). In Supplementary Fig. 14d, the experimental data is replotted where we divided the concentrations into three regions. < 200 aM region where band gap opening is dominant, >200 aM and <1nM region where the charge transfer becomes significant in addition to the band gap and >1nM region where the charge transfer is dominant while DNA adsorption saturation on graphene takes place.

Modeling of Dirac point shift for band gap opening

The shift is directly obtained from $\Delta V_{\mathrm{D}} = \frac{\mathrm{e}N_{\mathrm{DNA}}^{\mathrm{unscreened}}}{C_{\mathrm{T}}}$ without including any effect from bandgap. However, for the ultralow concentrations (e.g., 2 aM), the shifts are too small to be noticed without including the effects from bandgap opening (Supplementary Fig. 15c-d and Supplementary Fig. 14a-d).

The carrier charge density change of graphene due to a bandgap change after adding DNA molecules can be obtained from $\Delta n_{\text{bandgap}} = \frac{1}{\text{e}\rho}(\frac{1}{\mu^{\text{DNA}}} - \frac{1}{\mu^{\text{no DNA}}})$, where *e* is the elementary charge, and ρ is the resistivity. Wang *et al*.[1] showed that electronic mobility (μ) in graphene decreases with increasing bandgap where mobility is obtained empirically from $\mu = 0.114x10^4 E_g^{-\frac{3}{2}}$ (E_g in eV and μ in $cm^2\ V^{-1}\ s^{-1}$). Since the bandgap changes are local, the global (macroscopic) change in the charge carrier density is estimated by $\Delta n_{\text{global}} = b\ \Delta n_{\text{bangap}}$, where *b* is the area fraction of the affected regions. For the 2 aM concentration, assuming a bandgap change from 0.4224 eV to 1.7641 eV in 10^{-7}% of the crumpled graphene where each DNA nucleotide affects at least an area of 39.9 nm^2 (see the calculation below in the next section), the shift is noticeable and is estimated to be ~5mV (using $\Delta V_{\text{D}} = \frac{\text{e}\Delta n_{\text{global}}}{C_{\text{T}}}$) as shown in Supplementary Fig. 15e. 39.9 nm^2/nucleotide (an area with a diameter of 7.1nm) is relatively large compared to the size of a single DNA nucleotide. To ensure the bandgap change is indeed long-range, we investigated the effect of graphene size on the bandgap opening due to a single DNA base. As shown in Supplementary Fig. 16, we first considered one unit cell (width of ~12 and length of ~12 Å) and computed the bandgap of crumpled graphene in the presence of A-base (with the orientation 1 as indicated in Supplementary Table 3). Then, keeping the single A-base, we increased the number of the crumpled graphene unit cells to 2, 3 and 4. For example, a 4-unit cell is a supercell with a width of ~12 Å and a length of ~48 Å. Note that the width of all the cells is ~12 Å and only the length is varied. Based on this study, we observe that the bandgap for the case of 4-unit cell is about 60% of that of a single unit cell which is still a significant bandgap value. This supports our hypothesis that the local change of bandgap is long-range and influences the electronic properties of graphene globally.

Crumpling graphene by introducing 1D periodic ripples is found to produce a bandgap opening[2-4]. The opening of bandgap is attributed to the change in graphene curvature introducing quantum confinement with distinct electronic structures compared to the pristine/flat graphene[3]. In pristine graphene, the C-C bond length is ~1.41 Å for all carbon atoms with an angle of 120° which results in sp^2 hybridization. When graphene is crumpled, the bond length and the angles vary across the graphene. The optimized structure that we obtained using DFT shows that the C-C bond length has a value of 1.41-1.55 Å depending on the local curvature. In addition, the angles between the carbon atoms is found to be either 120° or 110°. The bond length of 1.55 Å and angle of 110° resemble the structure for sp^3 hybridized C atoms[5,6]. Thus, crumpled graphene contains sp^3 and sp^2 hybridization between C atoms. This is expected since the C atoms are not in the same plane due to crumpling. Further, the partial density of states of the pristine graphene (see Supplementary Figure 20) shows the px and pz orbitals having sp^2 hybridization. By analyzing the partial density of states of the crumpled graphene (see Supplementary Figure 20), we observe overlapping between all three p orbitals (i.e., px, py and pz) and between only two of them at a given energy level supporting the presence of both sp^3 and sp^2 hybridizations. Introducing sp^3 bonds produces strongly localized hybridization[7-8] resulting in a bandgap opening. It should be noted that changing the degree of hybridization (i.e., electronic orbitals overlap) via changing the bonding interactions (*e.g*. bond length and angle) is found to play a significant role in controlling the conduction and valence energy band levels which leads to altered bandgaps in different materials[9-13].

Calculation of area needed for low number of molecules

$\Delta n_{\text{bandgap}}(\text{local}) = \frac{1}{\text{e}\rho}(\frac{1}{\mu^{DNA}} - \frac{1}{\mu^{no\ DNA}})$, $\mu = 0.114 \times 10^4 E_g^{-\frac{3}{2}}$

$$\mathrm{e}\Delta n_{\mathrm{bandgap}}(\mathrm{local}) = \frac{1}{\sim 100\ \Omega}\left(\frac{1.7641^{\frac{3}{2}}}{0.114x10^4} - \frac{0.4224^{\frac{3}{2}}}{0.114x10^4}\right) Vs\ cm^{-2} = 1.815 \times 10^{-5}\ C\ cm^{-2}$$

$$\Delta V(\mathrm{local}) = \frac{\mathrm{e}\Delta n(\mathrm{local})}{C\ (\mathrm{local\ in\ concave})} = \frac{1.815\times10^{-5} C\ cm^{-2}}{0.16\times10^{-5}\ f\ cm^{-2}} = 1.1343 \times 10^4 V\ or\ 1.1343 \times 10^7 mV$$

To get 0.1 mV shift, $\mathrm{fraction} = \frac{0.1\ mV}{1.1343\times10^7 mV} = 8.8 \times 10^{-9}\ or \sim 10^{-7}$

DNA orientations in Ab Inito calculations

In orientation 1 and orientation 2 (see Supplementary Fig. 17 and Fig. 18), the plane of the base ring is parallel and perpendicular to the graphene surface, respectively. The plane of the base ring in orientation 3 is the same as in orientation 2. However, the nucleobase is rotated 180° as shown in Supplementary Fig. 17 and Fig. 18.

Since GW is computationally expensive, we only performed GW for orientation 1 on all the graphene surfaces and orientation 2 on the crumpled armchair graphene. Comparing the DFT and GW bandgaps of crumpled graphene, we note that the DFT bandgaps for orientations 1 and 2 are almost identical. However, the GW bandgaps are different for different orientations. To understand the interactions of the different orientations, we computed the interfacial charge density. The charge density difference of the system (i.e., graphene and nucleobase system), $\Delta\rho_s$, is defined as

$$\Delta\rho_s = \rho_s - \rho_g - \rho_n$$

where ρ_s is the charge density of the full system including both the crumpled graphene and the nucleobase in the unit cell, ρ_g is the charge density obtained by simulating the crumpled graphene without the nucleobase, and ρ_n is the charge density obtained by simulating the nucleobase without the crumpled graphene. $\Delta\rho$ _s represents the interfacial charge density due to the adsorption of the nucleobase onto the crumpled graphene. As shown in Supplementary Fig. 19, when the nucleobase is placed parallel to the crumpled graphene, the interfacial charge density is distributed along the nucleobase atoms. However, when the nucleobase is perpendicular to the crumpled graphene, the charge density is predominantly on the lower edge of the nucleobase experiences. Therefore, parallel orientation has a larger interfacial area with more Coulombic interactions (all atoms in the nucleobase directly interact with the crumpled graphene) compared to the perpendicular orientation (only the atoms towards the lower edge of the nucleobase have a direct interaction with the graphene surface). The different charge distributions and stacking lead to different electronic interactions for each orientation[14,15]. Further, Supplementary Fig. 19 shows the local electrostatic and local Hartree potential of each orientation in the non-periodic direction (c-direction shown in Supplementary Fig. 19a). At the interface, for orientation 1, there is a high energy barrier compared to orientation 2, which results in a higher bandgap for orientation 1. Different interactions in these orientations lead to different band gaps as captured by the GW method which accounts for Coulombic interactions using a many-body approach[16,17].

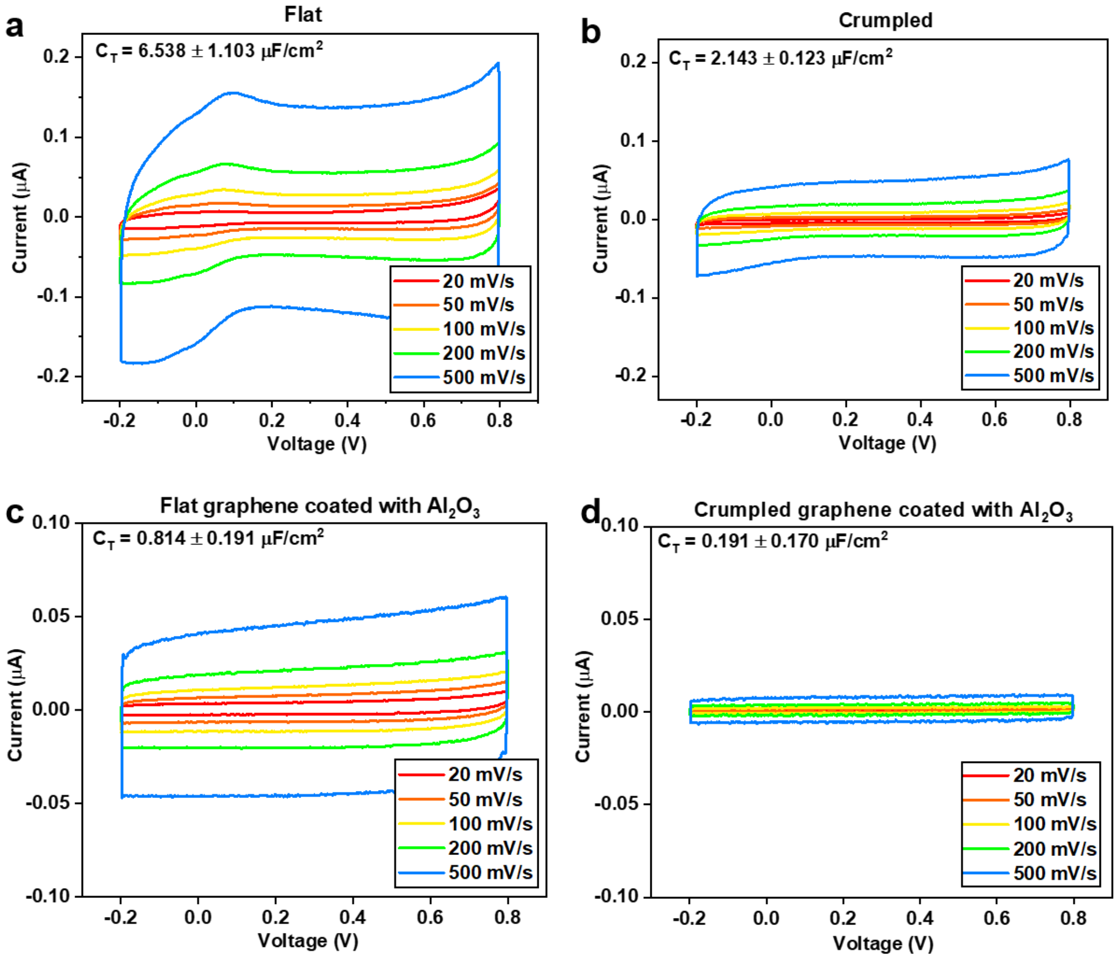

Supplementary Figure 23. EDL capacitance of graphene. **a**, the capacitance measurement of flat graphene. **b**, the capacitance measurement of crumpled graphene. The capacitance of flat graphene was about 3 times larger than the capacitance of crumpled graphene. **c**, **d**, capacitance measurement with atomic layer deposition (ALD). 5 nm of Al2O3 was deposited on both flat and crumpled graphene by (ALD) to reduce the leakage current. Flat graphene still showed about 4 times larger capacitance crumpled graphene. Both experiments showed that most of the capacitance change was attributed to the modulated screening length due to the nanoscale morphology.

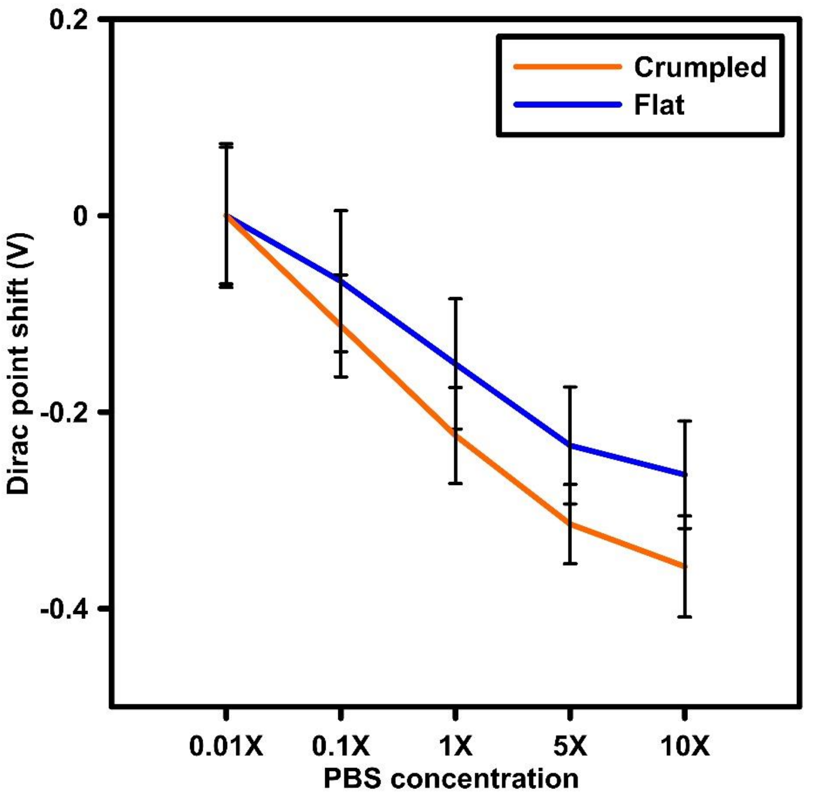

Supplementary Figure 24. Dirac point shift with four different concentrations of PBS buffer solution. *n*=5, mean ± std.

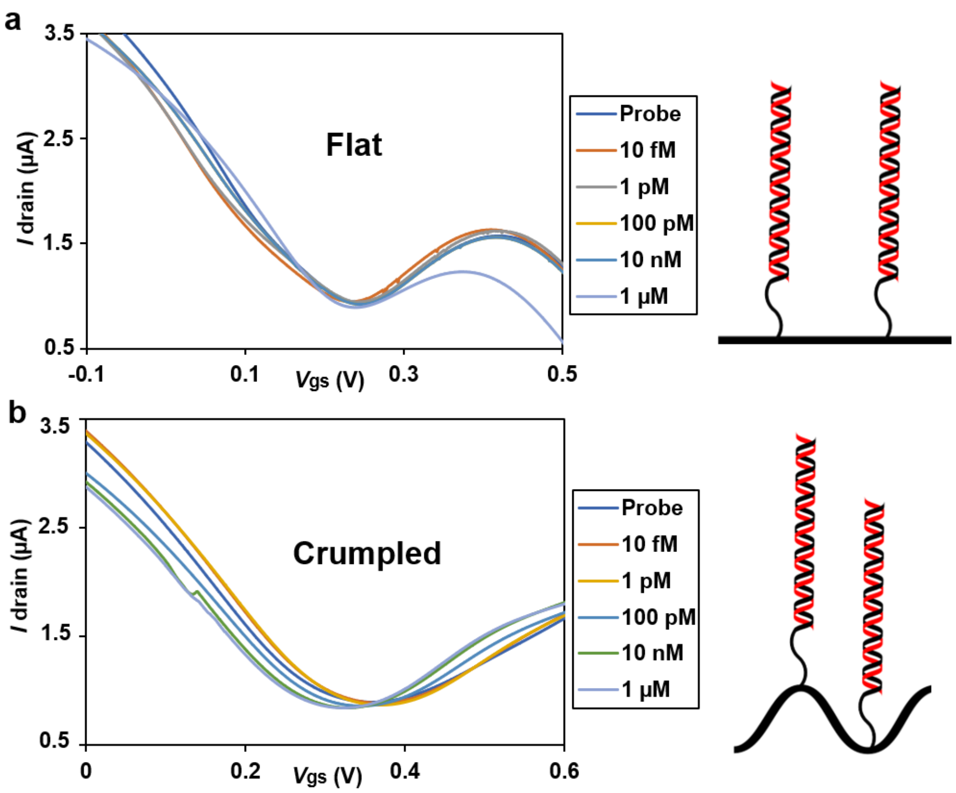

Supplementary Figure 25. *I*-*V* curve of charge layer distance experiment. **a,** While flat graphene FET does not show significant Dirac point shift, **b**, crumpled graphene FET showed left shift of IV curves. It supports the hypothesis of Debye length or EDL length change crumpling the graphene into nanoscale morphology.

References

1. Wang, J. Y., Zhao, R. Q., Yang, M. M., Liu, Z. F. & Liu, Z. R. Inverse relationship between carrier mobility and bandgap in graphene. *J. Chem. Phys.* **138**, doi:10.1063/1.4792142 (2013).
2. Bai KK, Zhou Y, Zheng H, Meng L, Peng H, Liu Z, Nie JC, He L. Creating one-dimensional nanoscale periodic ripples in a continuous mosaic graphene monolayer. Physical review letters. 2014 Aug 18;113(8):086102.
3. Lee JK, Yamazaki S, Yun H, Park J, Kennedy GP, Kim GT, Pietzsch O, Wiesendanger R, Lee S, Hong S, Dettlaff-Weglikowska U. Modification of electrical properties of graphene by substrate-induced nanomodulation. Nano letters. 2013 Jul 19;13(8):3494-500.
4. Lim H, Jung J, Ruoff RS, Kim Y. Structurally driven one-dimensional electron confinement in sub-5-nm graphene nanowrinkles. Nature communications. 2015 Oct 23;6:8601.
5. Sofo JO, Chaudhari AS, Barber GD. Graphane: A two-dimensional hydrocarbon. Physical Review B. 2007 Apr 10;75(15):153401.
6. Boukhvalov DW, Katsnelson MI, Lichtenstein AI. Hydrogen on graphene: Electronic structure, total energy, structural distortions and magnetism from first-principles calculations. Physical Review B. 2008 Jan 22;77(3):035427.
7. Abrasonis G, Gago R, Vinnichenko M, Kreissig U, Kolitsch A, Möller W. Sixfold ring clustering in s p 2-dominated carbon and carbon nitride thin films: A Raman spectroscopy study. Physical Review B. 2006 Mar 24;73(12):125427.
8. Zhang Y, Shang J, Fu W, Zeng L, Tang T, Cai Y. A sp 2+ sp 3 hybridized carbon allotrope transformed from AB stacking graphyne and THD-graphene. AIP Advances. 2018 Jan 30;8(1):015028.
9. Miglio A, Heinrich CP, Tremel W, Hautier G, Zeier WG. Local bonding influence on the band edge and band gap formation in quaternary chalcopyrites. Advanced Science. 2017 Sep;4(9):1700080.
10. Jaffe JE, Zunger A. Theory of the band-gap anomaly in AB C 2 chalcopyrite semiconductors. Physical Review B. 1984 Feb 15;29(4):1882.
11. Khoa DQ, Nguyen CV, Bui LM, Phuc HV, Hoi BD, Hieu NV, Nha VQ, Huynh N, Nhan LC, Hieu NN. Opening a band gap in graphene by C–C bond alternation: a tight binding approach. Materials Research Express. 2019 Jan 9;6(4):045605.
12. Prasanna R, Gold-Parker A, Leijtens T, Conings B, Babayigit A, Boyen HG, Toney MF, McGehee MD. Band gap tuning via lattice contraction and octahedral tilting in perovskite materials for photovoltaics. Journal of the American Chemical Society. 2017 Aug 4;139(32):11117-24.
13. Peng X, Velasquez S. Strain modulated band gap of edge passivated armchair graphene nanoribbons. Applied Physics Letters. 2011 Jan 10;98(2):023112.
14. Park C, Atalla V, Smith S, Yoon M. Understanding the Charge Transfer at the Interface of Electron Donors and Acceptors: TTF–TCNQ as an Example. ACS applied materials & interfaces. 2017 Aug 2;9(32):27266-72.
15. Fernández AC, Castellani NJ. Dipole moment effects in dopamine/N-doped-graphene systems. Surface Science. 2020 Mar 1;693:121546.
16. Hybertsen MS, Louie SG. Electron correlation in semiconductors and insulators: Band gaps and quasiparticle energies. Physical Review B. 1986 Oct 15;34(8):5390.
17. Johnson KA, Ashcroft NW. Corrections to density-functional theory band gaps. Physical Review B. 1998 Dec 15;58(23):15548.
